# Lipocalin 2 Facilitates the Initial Compromise of the Blood–Brain Barrier Integrity in Chronic Cerebral Hypoperfusion

**DOI:** 10.1111/cns.70438

**Published:** 2025-05-14

**Authors:** Qianqian Nie, Liren Zhang, Zhengsheng Gu, Yuchao Li, Xiaoying Bi

**Affiliations:** ^1^ Department of Neurology Changhai Hospital, Naval Medical University Shanghai China; ^2^ Department of Nuclear Medicine Changhai Hospital, Naval Medical University Shanghai China

**Keywords:** blood–brain barrier, chronic cerebral hypoperfusion, endothelial‐mesenchymal transition, Lipocalin 2, vascular cognitive impairment

## Abstract

**Aims:**

Vascular cognitive impairment (VCI) is primarily attributed to vascular risk factors and cerebrovascular disease. Chronic cerebral hypoperfusion (CCH) initiates in the early stages of VCI and contributes to blood–brain barrier (BBB) disruption. Nevertheless, the precise molecular mechanisms underlying this process remain elusive and warrant further investigation.

**Methods:**

The Bilateral Carotid Artery Stenosis (BCAS) model was employed to simulate CCH, and the permeability of the BBB in the mouse frontal cortex was assessed at various time points post‐operation. Transcriptome sequencing was conducted to identify differentially expressed genes (DEGs), which were then intersected with cortical gene chip data from human vascular dementia cases. Key genes identified through this analysis were subsequently measured in the serum of VCI patients and correlated with cognitive performance scores. Additionally, in vivo experiments were conducted to validate the influence of these key genes on BBB endothelial‐mesenchymal transition (EndMT) and peripheral neutrophil infiltration.

**Results:**

Time‐course studies investigating BBB disruption revealed an increase in BBB permeability in the frontal cortex of mice on day 14 following BCAS, a stage at which the mice had not yet exhibited cognitive impairment. Subsequent sequencing analyses, integrated with human cortical gene expression profiles, identified Lipocalin 2 (LCN2) as a pivotal gene involved in mediating inflammatory responses and cell migration. Clinical studies have demonstrated that LCN2 is upregulated in the serum of patients with VCI and exhibits a significant negative correlation with Montreal Cognitive Assessment (MOCA) scores. Downregulation of LCN2 leads to a reduction in EndMT markers and peripheral neutrophil infiltration, as well as significant enhancements in learning and memory in BCAS mice through modulation of the MEK/ERK signaling pathway.

**Conclusion:**

This study elucidates the temporal characteristics and key molecular mechanisms underlying BBB disruption in the frontal cortex during CCH, thereby identifying novel potential targets for the early diagnosis and treatment of VCI.

## Graphical abstract

We identified a pivotal gene LCN2, which may affect BBB permeability and mediate peripheral neutrophil infiltration by inducing EndMT in the early stage of CCH. Furthermore, LCN2 is markedly overexpressed in the serum of patients with VCI, suggesting its potential utility as a biomarker.

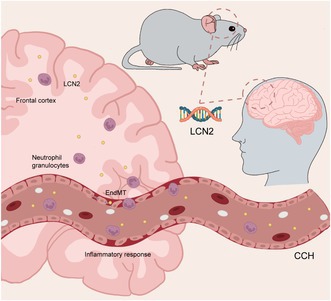

## 1 Introduction

Vascular cognitive impairment (VCI) is a large group of syndromes ranging from mild cognitive impairment to dementia caused by cerebrovascular disease and its risk factors, with a clinical incidence second only to Alzheimer's disease (AD), accounting for about 20% of all dementia types [1, 2, 3]. Current research on biomarkers of VCI predominantly concentrates on its severe manifestation of vascular dementia (VaD), lacking early warning indicators capable of capturing the pathological process before cognitive impairment [4, 5].

Chronic cerebral hypoperfusion (CCH) is recognized as a pivotal early event in the pathological progression of VCI [6, 7], initiating a cascade of neuroimmune and inflammatory responses [8, 9]. Recent findings indicate that CCH‐induced disruption of the blood–brain barrier (BBB) not only occurs prior to the onset of clinical symptoms but also functions as a dynamic nexus that facilitates cognitive decline [10, 11]. It is important to highlight that while current research has clarified the mediating role of endothelium‐mesenchymal transformation (EndMT) in BBB impairment, it has yet to elucidate how the spatiotemporal heterogeneity of EndMT influences cognitive deterioration across various brain regions, which greatly constrains the identification of potential early intervention targets [12, 13, 14].

Lipocalin 2 (LCN2) is a secreted protein belonging to the lipocalin superfamily, which can mediate various biological processes such as inflammatory response, innate immune response, cell migration and differentiation, energy metabolism, etc. [15, 16]. LCN2 has been demonstrated to be significantly correlated with increased BBB permeability in acute cerebral ischemia [17, 18]; however, its regulatory mechanism concerning BBB integrity in the context of CCH remains to be elucidated.

In this study, we employed transcriptome sequencing in conjunction with a case–control analysis of VCI to systematically investigate the temporal and spatial progression of BBB disruption in the frontal cortex during CCH. Our findings reveal the dynamic expression characteristics of LCN2 in this process and demonstrate its role in activating the MEK/ERK signaling pathway to induce EndMT. In conclusion, we have identified a novel liquid biomarker with potential utility for the early detection of VCI progression, offering a promising new avenue to address the current challenges associated with delayed diagnosis.

## 2 Materials and Methods

### 2.1 Animals

All experiments involving mice were approved by the Animal Ethics Committee of the First Affiliated Hospital of the Naval Medical University (Changhai Hospital) and complied with all ethical regulations regarding animal testing and research. C57Bl/6 mice were housed in a standard animal house at a temperature of 22°C ± 2°C and a humidity of 55% ± 5%, with ad libitum access to food and water. All experiments involving mice comply with the Animal Research: Reporting of in Vivo Experiments (ARRIVE) guidelines. Animal suffering was minimized during the experiment. Animal numbers: each group satisfies *n* ≥ 6.

### 2.2 Bilateral Carotid Artery Stenosis Procedure and Pharmaceutical Intervention

Bilateral carotid artery stenosis (BCAS) surgery was performed as described previously [19]. Microcoils (inner diameter: 0.18 mm) were purchased from Sawane Spring Co (Sawane, Japan). After anesthetizing the mice with sodium pentobarbital, the bilateral common carotid arteries were exposed through a median neck incision. A microcoil was wrapped around the common carotid artery on one side. After an interval of 30 min, the other microcoil was wrapped around the contralateral common carotid artery in a symmetrical position [20, 21, 22]. The sham‐operated group was operated on in the same way as the operated group, except that no microcoils were placed.

U0126 treatment (109511‐58‐2, MCE): mice were injected intraperitoneally with 10 mg/kg U0126 daily from days 7 to 13. rLCN2 (1 mg/kg, 2 μl; Cat. No. CM17, novoprotein) was injected into the frontal cortex of mice on day 7 according to the standard procedure for lentiviral injection.

### 2.3 Cerebral Blood Flow Measurement

Cortical CBF was monitored using a laser speckle flow imaging technique 1 day after BCAS. Mice were anesthetized and placed on a stereotaxic frame to shave the head, disinfected with iodophor, and the skull exposed. The fascia attached to the skull was removed as much as possible, and 0.9% saline was added to maintain the fluid level. Cerebral blood flow images were acquired with a laser speckle flow imaging instrument (SIMOPTO, China). All procedures were performed under double‐blind conditions.

### 2.4 Evaluation of BBB Permeability

Evans blue stain binds to serum albumin immediately after injection into the bloodstream. Plasma albumin does not cross the BBB under normal physiological conditions, so using spectrophotometry to determine the accumulation of Evans blue stain is a simple and reliable method of assessing the permeability of the BBB [23, 24]. Briefly, mice were injected intraperitoneally with 2% Evans blue solution (10 mL/kg). The stain was allowed to circulate for 4 h. Subsequently, 50 mL of ice‐cold PBS was perfused transcardially. Brain tissues were removed for homogenization, and a supernatant was taken. An equal amount of 50% trichloroacetic acid was added, and the incubation was carried out overnight at 4°C. It was analyzed by spectrophotometry at 620 nm and quantified according to a standard curve.

### 2.5 RNA Sequencing and Differentially Expressed Gene Analysis

Mouse brain frontal cortex tissue was removed and fully immersed in RNALater RNA Stabilization Reagent. The tissues were lysed and homogenized to extract total RNA, and RNA sequencing was performed according to previously reported procedures [25]. Strict quality control of RNA samples is carried out, which mainly includes concentration quality control, fragment integrity quality control, and purity quality control. Gene expression profiles of the frontal and temporal cortex of human VaD and non‐demented controls (Control) GSE122063 were obtained from the Gene Expression Omnibus (GEO) database. Differentially expressed genes (DEGs) were analyzed using the edgeR package. A gene was considered significantly downregulated if *p* < 0.05 and log2 fold change ≤ −1. Genes were considered upregulated if *p* < 0.05 and log2 fold change ≥ 1.

### 2.6 Bioinformatics Analysis

Weighted gene correlation network analysis (WGCNA) was performed on the raw transcript dataset of GSE122063 using WGCNA package v1.69 to calculate the correlation between different gene modules and VaD. Check scale‐free topology scale R^2^ = 0.8. Cytoscape software v3.91 was used to visualize the co‐expression network and identify the key genes in the module. The STRING (https://string‐db.org/) database, Metascape (https://metascape.org/) database, and GeneMANIA (http://genemania.org/) online database were used to predict the biological functions of the DEGs, and a protein–protein interaction (PPI) network was constructed. Gene Ontology (GO) functional enrichment analysis and Kyoto Encyclopedia of Genes and Genomes (KEGG) pathway were performed in the DAVID database.

### 2.7 Western Blotting

The protein concentration was determined by Western blotting. In brief, mouse brain tissues were removed and lysed by grinding on ice with RIPA buffer supplemented with protease inhibitors. Protein was quantified using the BCA protein assay kit according to the manufacturer's instructions. SDS‐PAGE separated proteins and subsequently transferred them to NC membranes (Millipore). The membranes were blocked in Rapid Blocking Solution for 15 min at room temperature, washed three times using PBST, and incubated overnight in a 4° refrigerator with specific first antibodies. The blots were incubated with anti‐rabbit or anti‐mouse secondary antibodies the following day. The antibodies used were as follows: LCN2 (ab318209, 1:1000, Abcam); N‐cadherin (22018‐1‐ap, 1:2000, Proteintech); Vimentin Monoclonal Antibody (60330‐1, 1:20,000, Proteintech); VE‐cadherin (27956‐1‐AP, 1:1000, Proteintech); Phospho‐ERK1/2 (Thr202/Tyr204) Polyclonal Antibody (28733‐1‐AP, 1:1000, Proteintech); ERK1/2 Polyclonal Antibody (11257‐1‐AP, 1:2000, Proteintech); Phospho‐MEK1/2 (Ser217/221) (41G9) Rabbit mAb (9154, 1:1000, CST); MEK1/2 (L38C12) Mouse mAb (4694; 1:1000;CST); Albumin Polyclonal Antibody (16475‐1‐AP, 1:5000, Proteintech). Membranes were exposed to light‐sensitive film and quantified using ImageJ software.

### 2.8 Immunofluorescence

The brains of mice were collected after cardiac perfusion with paraformaldehyde for paraffin‐embedded sections. Sections were deparaffinized, antigenically repaired, and blocked for 1 h, then incubated with primary antibody at 4°C overnight. After washing with PBST, the secondary antibody was incubated in a darkened box at room temperature for 1 h, stained with DAPI staining solution at room temperature in the dark for 10 min, and washed three times with PBS for 5 min each time. Observations were performed using a high‐resolution inverted fluorescence microscope, and a suitable fluorescent light source was selected and photographed. Quantitative analysis of fluorescence images was performed using Image J software. Antibodies used were as follows: LCN2 (ab318209, 1:200, Abcam); LY6G (GB12229‐50, 1:200, Servicebio).

### 2.9 Lentiviral Transfection

The Sh‐LCN2 and Sh‐NC lentiviruses were purchased from Jikai Gene (Shanghai Genechem Co. Ltd.). Sh‐LCN2 silencing sequence: 5′‐CGCTACTGGATCAGAACATTT‐3′; Sh‐NC sequence: 5′‐TTCTCCGAACGTGTCACGT‐3′. Mice were fixed to a stereotaxic frame on the seventh day after BCAS surgery, and the skull was exposed by cutting the skin of the head. Injections were made into the frontal cortex (Coordinates were as follows: *X* = −1.0, *Y* = 1.5, *Z* = 2.4, with Bregma serving as the 0 point for *X* and *Y*). The viral titer was established at 1 × 10^8^ transducing units per milliliter, with an administration of 2 μL per mouse. The needle was left in place for an additional 5 min after completion of the injection and then slowly removed. The animals were grouped as follows: (1) sham: The sham‐operated group was operated in the same way as the BCAS‐operated group, except that no microcoils were placed. BCAS 14d: (1) Con: Blank control group with no treatment except BCAS surgery. (2) Sh‐NC: BCAS surgery was performed, and negative control lentivirus was injected. (3) Sh‐LCN2: BCAS surgery was performed, and LCN2 silencing lentivirus was injected.

### 2.10 Morris Water Maze

Morris water maze (MWM) is an experimental method for studying brain learning and memory [26]. The test procedure mainly consists of two parts: localization navigation test and spatial exploration test. The water temperature of the water maze was maintained at 24°–25°, and the water was dyed white with white, non‐toxic, edible coloring. The pool was divided equally into four quadrants, and a white platform was set underwater in the second quadrant. The height of the platform was about 0.5 cm–1 cm below the horizontal plane, and the platform position was kept fixed during the test. The training was conducted 1 week before the formal experiment, three times a day. The mice were allowed to explore freely in the water for 1 min, and if the platform was not found for more than 1 min, the mice were guided to the platform and allowed to stay on the platform for 15 s. The location navigation experiment was performed on days 30–34 after BCAS, and the time to find the platform and the swimming path of the mice were recorded. On day 35, the underwater hidden platform was removed, and the mice were placed into the pool from the fixed placement point. The number of times the mice crossed the previous platform position within 1 min, the swimming path was recorded, and the whole process was videotaped for subsequent analysis.

### 2.11 Case–Control Study

This study, registered in the Chinese Clinical Trial Registry with registration number ChiCTR2000030614, was conducted according to the revised Declaration of Helsinki. Written informed consent was obtained from all participants. The First Affiliated Hospital Ethics Committee of Naval Medical University approved the study. The primary study subjects were patients with the first diagnosis of VCI in the outpatient clinic or ward of the Department of Neurology, Changhai Hospital, matched with non‐cognitively impaired elderly control patients in the same period.

### 2.12 Inclusion Criteria

(1) Age > 18 years; (2) evaluated by the 2024 edition of the Chinese Guidelines for the Diagnosis and Treatment of VCI: (1) presence of cognitive impairment (including the patient's subjective self‐report or informant's report of impairment of cognitive function), and the presence of ≥ 1 cognitive domain impairment as measured by neuropsychology. (2) Cognitive function was assessed using the MoCA scale with a MoCA < 26 points. (3) Hachinski ischemic score (HIS) score of ≥ 7 on the index of ischemia scale. Presence of one or more risk factors for cerebrovascular disease (e.g., hypertension, diabetes mellitus, hyperlipidemia, coronary heart disease, etc.).

### 2.13 Study Groups

1. VCI group (with vascular risk factors + with imaging manifestations + MoCA score < 26).
2. Control group (with vascular risk factors + MoCA score ≥ 26).

### 2.14 Exclusion Criteria

1. Cognitive impairment caused by other neurodegenerative rather than vascular factors, such as AD, Parkinson's disease dementia, frontotemporal lobe dementia, etc.
2. Previous history of cognitive impairment or other conditions affecting cognitive function.
3. Exclusion of emotional problems such as severe anxiety and depression.
4. Inability to cooperate with completing the neuropsychological scale assessment.
5. Serious cardiac, hepatic, or renal insufficiency or malignant tumors affect the life expectancy of patients. Propensity Score Matching (PSM) was employed to balance the baseline characteristics, utilizing a caliper value of 0.1; the statistical power is 0.86.

### 2.15 Enzyme‐Linked Immunosorbent Assay

Serum LCN2 levels in mice and humans were analyzed using the LCN2 ELISA kit (WELLBIO, shanghai). The trial was conducted according to the manufacturer's instructions. Final results were recorded with an enzyme‐linked immunosorbent assay (ELISA) plate detector at 450 nm. The ELISA standard curve covering 0.1–200 ng/mL (*r* ^2^ = 0.9998) with *n* = 6.

### 2.16 Statistical Analysis

For data analysis, SPSS version 26.0 (SPSS Inc., Chicago, IL, USA) was utilized. Measurement data are expressed as mean ± SE (all from ≥ 6 independent experimental replicates). The normality of continuous variables was assessed using the Shapiro–Wilk test. Data following a normal distribution are reported as mean ± standard deviation (SD), whereas non‐normally distributed data are represented by the median and interquartile range. For comparisons between groups with normally distributed data, an independent samples *t*‐test was employed, and one‐way analysis of variance (ANOVA) was utilized for comparisons involving multiple groups. Categorical variables are expressed as frequency (percentage), with group comparisons conducted using the chi‐square (*χ* ^2^) test. Ranked data were analyzed using the Mann–Whitney *U* test or the Kruskal–Wallis *H* test. Correlation analysis was performed using the Pearson correlation coefficient. PSM was applied to control for confounding factors such as age and years of schooling. A *p*‐value of less than 0.05 was considered indicative of statistical significance.

## 3 Results

### 3.1 BBB Integrity Measurement in the Frontal Cortex of CCH Model Mice

**FIGURE 1:**
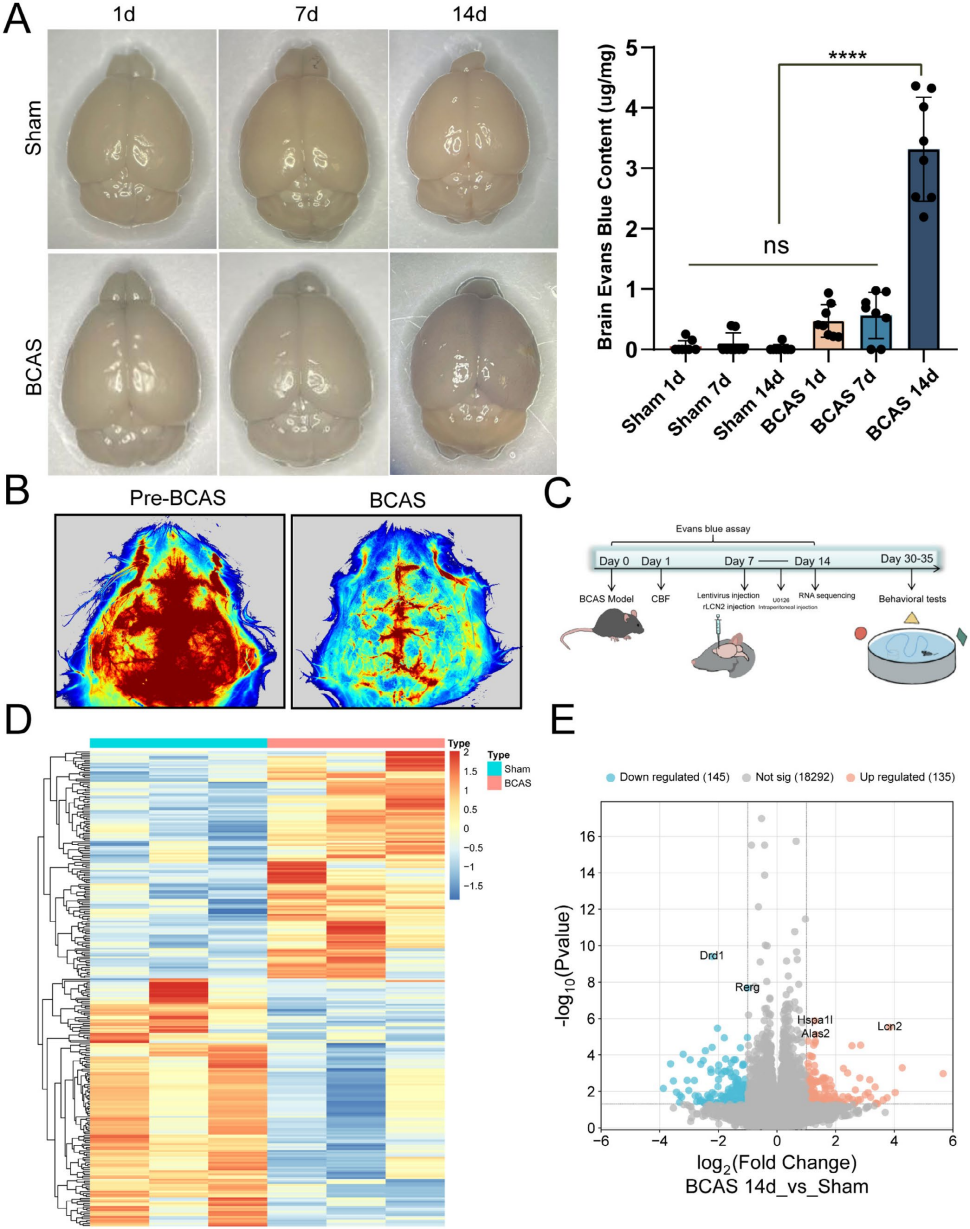
BBB permeability detection and transcriptome sequencing analysis. (A) Blood–brain barrier permeability assay: Evans blue staining showed BBB leakage on days 1, 7, and 14 after BCAS (*n* = 8). Quantitative analysis showed that brain Evans blue content was significantly higher in the BCAS 14d group compared to the other groups (****p < 0.0001). (B) Representative images of CBF before and after BCAS monitored by laser speckle cerebral blood flow imaging. (C) Experimental flow chart of the experimental process: Shows the time nodes of intervention measures, sample collection, molecular biological detection, and behavioral experiments. (D) Heat map of sham‐mRNA and BCAS 14d‐mRNA in transcriptome data microarray. The red part of the heat map indicates mRNA upregulation, and the blue part indicates mRNA downregulation. (E) Volcano plot of DEGs. Red dots represent significantly upregulated genes (*p* < 0.05 and log2 fold change ≥ 1), and blue dots represent significantly downregulated genes (*p* < 0.05 and log2 fold change ≤ −1).

To investigate the temporal properties of BBB damage after CBF reduction, we used Evans blue staining to detect BBB permeability in the frontal cortex of mice on different days after BCAS. The BCAS model is a well‐established animal model that mimics CCH, and it has been reported that the mice in this model have cognitive impairment 30 days after surgery [19]. The results showed that the content of Evans blue in the frontal cortex of mice on day 14 after BCAS was significantly higher than that in the sham‐operated group, the group on day 1 after BCAS, and the group on day 7 after BCAS (Figure 1A). The cerebral blood flow of mice before and after BCAS was monitored by laser speckle cerebral blood flow imaging, and the results were shown in Figure 1B, which indicated that the local cerebral blood flow in the frontal lobe was reduced after BCAS compared with that before surgery. These results demonstrated that the BBB permeability of the frontal cortex was significantly increased on the 14th day after BCAS, at which time the mice had not yet developed cognitive impairment. The overall experimental flow of this study is shown in Figure 1C.

### 3.2 BCAS 14d Mouse Frontal Cortex Transcriptome Sequencing and Analysis

To investigate the mechanism of frontal BBB damage on the 14th day after BCAS, we took the brain frontal cortex of mice from the BCAS group and the sham operation group on the 14th day after surgery for transcriptome sequencing. Bioinformatics analysis of the gene expression profiles obtained by sequencing yielded 280 DE mRNAs, including 135 upregulated and 145 downregulated mRNAs, which were visualized as heatmaps and volcano plots, as shown in Figure 1D,E.

### 3.3 Human Brain Microarray Data Mining and Functional Enrichment Analysis

**FIGURE 2:**
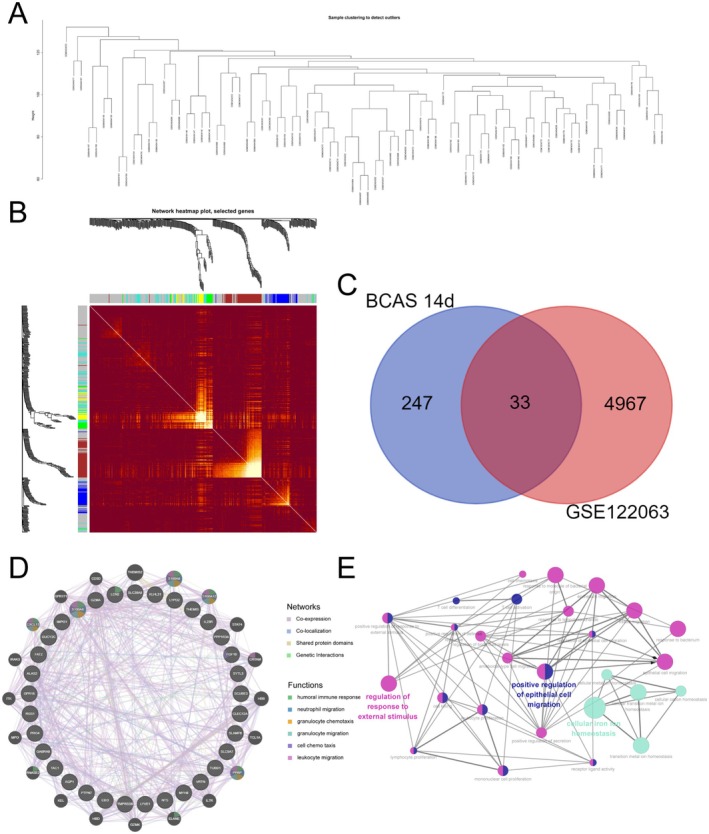
Acquisition of intersection DEGs and functional enrichment analysis. (A) Clustering dendrogram of WGCNA (*R* ^2^ = 0.83). (B) Heatmap of cluster modules by WGCNA: Green modules are significantly associated with the pathologic progression of vascular dementia. (C) Intersection Venn plot of VaD‐related genes and DEGs of BCAS 14 day. (D) GeneMANIA‐constructed gene–gene interaction network, with node size representing connectivity and edge color representing interaction type. (E) Cytohubba plug‐in visualized protein–protein interaction network and predicted biological functions.

We downloaded the cortical gene expression profile GSE122063 of VaD patients and non‐demented controls from the GEO database and performed WGCNA analysis to screen the gene module that was significantly associated with VaD. The clustering tree diagram and heatmap are shown in Figure 2A,B. Further, the VaD‐related module genes and BCAS model DEGs were intersected, and 33 intersection DEGs were obtained (Figure 2C). The genemania analysis revealed that these genes were mainly associated with humoral immune response, neutrophil migration, granulocyte chemotaxis, granulocyte migration, cell chemotaxis, and leukocyte migration (Figure 2D). Interaction networks were constructed using the Cytohubba plug‐in of Cytoscape. These genes were found to be mainly enriched in the epithelial cell migration, regulation of response to external stimulus, and cellular iron ion homeostasis pathways (Figure 2E).

### 3.4 Key Gene Analysis

**FIGURE 3:**
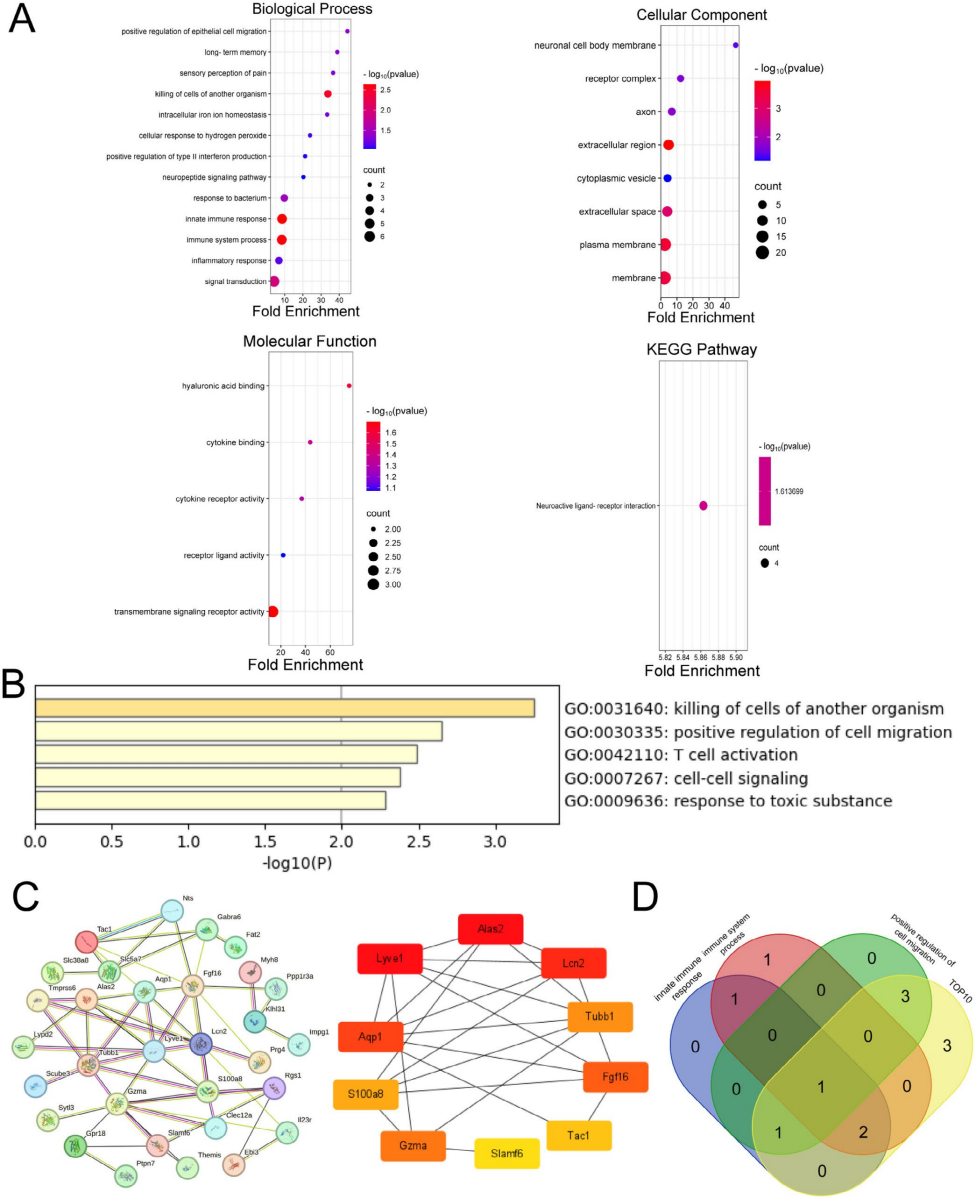
Mining key genes. (A) Bubble plots of GO and KEGG enrichment analysis of the DAVID database for biological processes, cellular components, molecular functions, and top enriched KEGG terms, respectively. (B) The top 5 biological processes enriched by Metascape. (C) PPI networks were visualized with the String database, and the top 10 genes were obtained by Cytoscape software. (D) Venn diagram of genes involved in major biological processes.

To identify key genes, we conducted an in‐depth analysis of the biological functions of 33 genes. GO and KEGG enrichment analyses, utilizing the DAVID database, revealed that the intersecting DEGs were predominantly associated with biological processes such as the innate immune response and immune system processes (Figure 3A). Analysis using the Metascape database indicated that these genes were primarily linked to the positive regulation of cell migration (Figure 3B). The 33 intersecting DEGs were submitted to the STRING database for PPI analysis, and the resulting interaction network was further analyzed using Cytoscape software to identify the top 10 candidate hub genes (Figure 3C). By intersecting the genes associated with the aforementioned biological pathways and the top 10 hub genes, we identified a key gene, LCN2. Our findings suggest that LCN2 may play a critical role in the inflammatory response and cell migration in CCH (Figure 3D).

### 3.5 LCN2 Expression and BBB Endothelial‐Mesenchymal Transition

**FIGURE 4:**
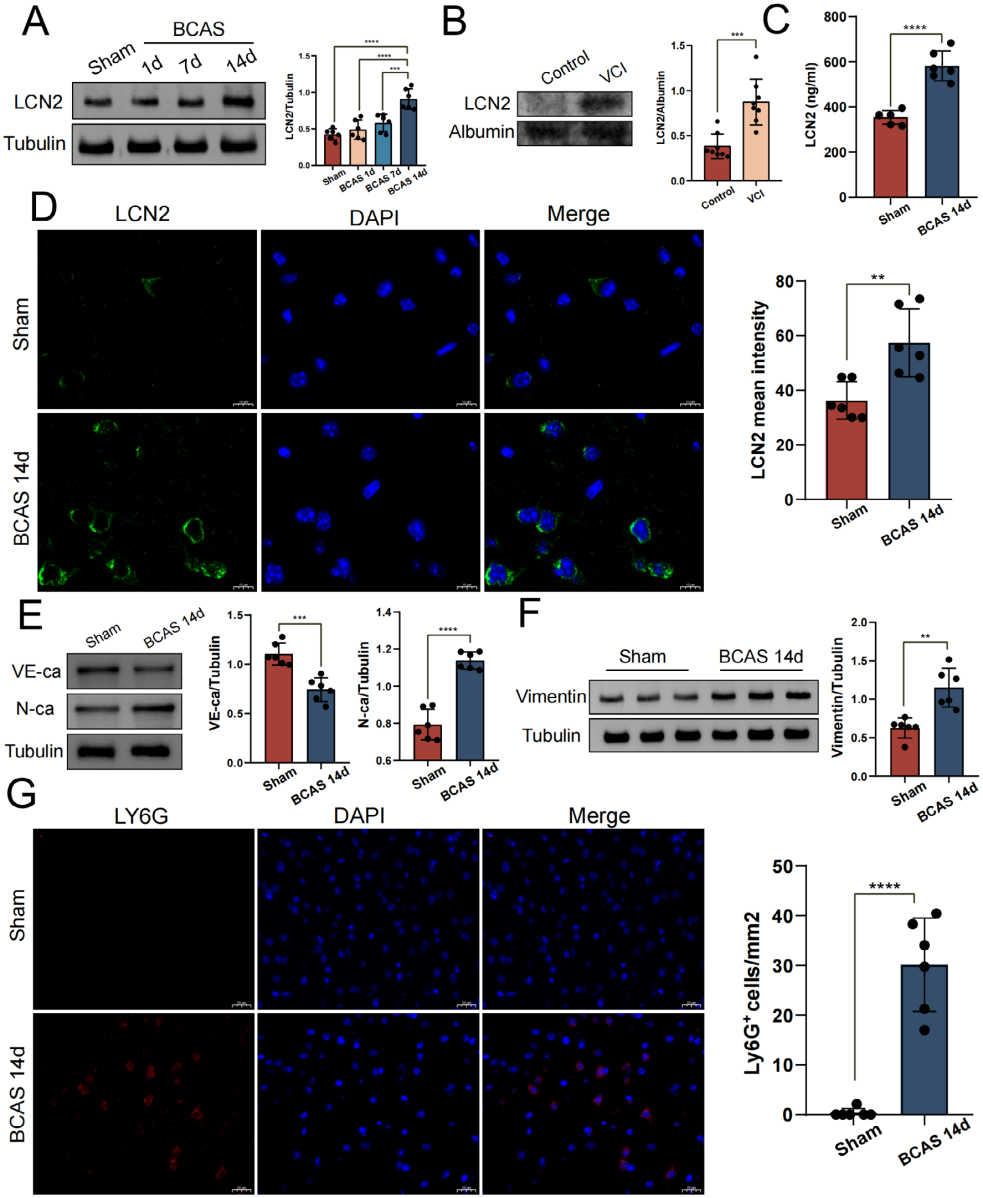
Validation of LCN2 expression and phenotypic study of BBB disruption. (A) Representative images and quantitative results of LCN2 expression at different days after BCAS (1d/7d/14d). (B) Representative images and quantitative results of LCN2 expression in control and VCI groups. (C) ELISA quantification of serum LCN2 concentration (ng/mL) in mouse (*n* = 6). (D) Immunofluorescence images and quantitative results of LCN2 in the frontal cortex of mice with green fluorescence showing LCN2 and blue fluorescence showing nuclei. (E) WB representative images and quantitative results of VE‐cadherins and N‐cadherins. (F) Representative images of vimentin in WB and quantitative results. (G) Immunofluorescence images and quantitative results of LY6G in mouse frontal cortex, red fluorescence shows LY6G, and blue fluorescence shows cell nuclei. Quantitative analysis was performed regarding the number of LY6G‐positive cells per square millimeter. **p < 0.01, ***p < 0.001, ****p < 0.0001.

A time‐course study of LCN2 expression revealed that LCN2 expression was significantly upregulated in the frontal cortex of mice on day 14 after BCAS compared with the sham group, consistent with the results of bioinformatics analysis (Figure 4A). Western blot detection of serum of patients in control and VCI group showed that the content of LCN2 in peripheral blood of patients in VCI group was significantly upregulated (Figure 4B). ELISA showed that the levels of LCN2 in the serum of the mice were also significantly upregulated (Figure 4C). Immunofluorescence staining showed that the fluorescence intensity of LCN2 in the frontal cortex of BCAS mice was significantly higher than that in the sham‐operated group, indicating that LCN2 was highly expressed in the mouse brain (Figure 4D). Further studies revealed that the mesenchymal markers neural cadherins (N‐cadherins) and vimentin were upregulated, and the adhesion junction protein vascular endothelial cadherins (VE‐cadherins) was downregulated in the frontal cortex of the BCAS 14d mice, indicating that an EndMT process occurs (Figure 4E,F). Bioinformatics analysis showed that LCN2 might be associated with the cell migration process, so we performed LY6G staining, and the results showed increased neutrophil infiltration in the frontal cortex of BCAS 14d mice (Figure 4G).

### 3.6 Effect of Silencing LCN2 on BBB

**FIGURE 5:**
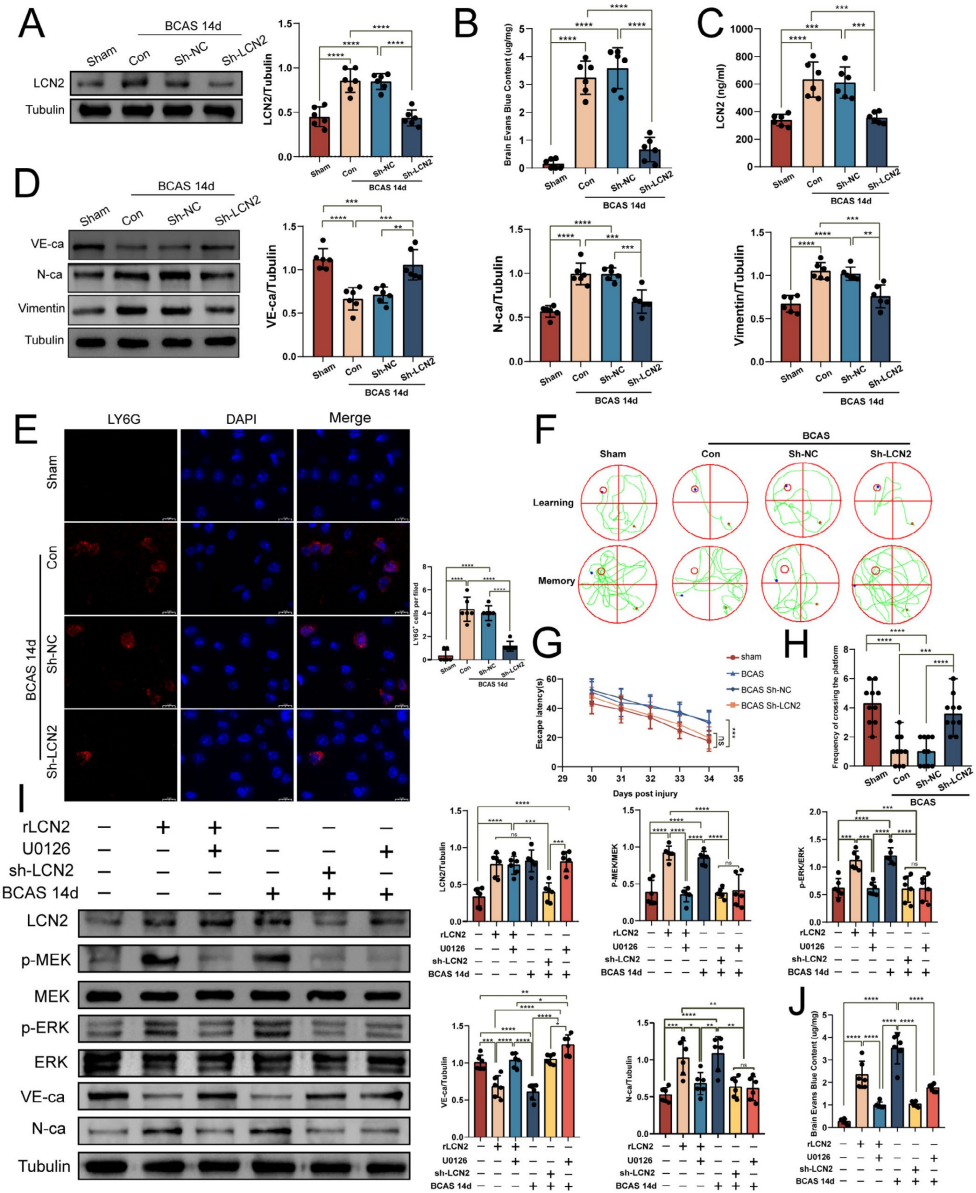
Effects of LCN2 knockdown on BBB and cognitive function. (A) Representative images and quantitative results of LCN2 expression. (B) Quantitative results of Evans blue stain content. (C) Quantitative results of LCN2 content in mouse serum. (D) Representative images and quantitative analysis results of endothelial‐mesenchymal transition markers. (E) Immunofluorescence image and quantitative analysis diagram of LY6G in the frontal cortex of mice, with red fluorescence showing LY6G and blue fluorescence showing nuclei. (F) Representative images of learning and memory in the Morris water maze. (G) Statistical analysis graph of escape latency. (H) Statistical analysis graph of the number of times mice crossed the platform in 1 min. (I) Western blot assay plots and quantitative analyses of the MEK/ERK signaling pathway and EndMT marker expression were conducted following the application of recombinant LCN2, Sh‐LCN2, and U0126 in both sham and BCAS14d groups, respectively. (J) Statistics of Evans blue content in the frontal lobes of mice after administration of recombinant LCN2, Sh‐LCN2, and U0126 in sham or BCAS groups, respectively. *p < 0.05, **p < 0.01, ***p < 0.001, ****p < 0.0001.

In order to examine the potential influence of LCN2 on the integrity of the BBB via the induction of EndMT, we introduced an LCN2‐silencing lentivirus into the frontal cortex of mice on the 7th day after BCAS surgery. Western blot analysis conducted 14 days post‐BCAS demonstrated that, in comparison to the group receiving a negative control lentivirus, the expression levels of LCN2 in the group treated with the silencing lentivirus were restored to baseline levels, showing no statistically significant difference from the sham‐operated group (Figure 5A). Evans blue staining demonstrated a marked decrease in Evans blue content in the frontal cortex of the Sh‐LCN2 group compared to the control and Sh‐NC groups (Figure 5B), indicating restoration of BBB integrity. Furthermore, ELISA results revealed a significant reduction in serum LCN2 levels following brain LCN2 silencing (Figure 5C), suggesting a correlation between serum and brain LCN2 concentrations. Importantly, in comparison to the control and Sh‐NC groups, the Sh‐LCN2 group exhibited downregulation of mesenchymal markers N‐cadherin and vimentin, alongside a significant upregulation of the adhesion junction protein VE‐cadherin, indicating a reversal of the EndMT process post‐LCN2 silencing (Figure 5D). Additionally, LY6G staining indicated that reduced LCN2 expression also diminished central infiltration of peripheral neutrophils (Figure 5E). We then performed a MWM test on the mice injected with the lentivirus on day 30 after BCAS surgery and found that the escape latency of the Sh‐LCN2 group was shorter than that of the blank control and vector groups (Figure 5F,G). In the space exploration test, mice in the Sh‐LCN2 group showed an increase in the number of crossing platforms (Figure 5H), and the difference was statistically significant. The above results suggest that silencing LCN2 may reduce BBB damage and improve the learning memory ability of mice by reversing the EndMT process.

### 3.7 MEK/ERK Pathway Mediates LCN2‐Induced EndMT and BBB Dysfunction

We examined the mediating role of the MEK/ERK signaling pathway to further elucidate the downstream mechanisms of LCN2. By administering rLCN2 into the frontal cortex of mice in the sham‐operated group, we observed an upregulation of phosphorylated MEK/ERK expression, a decrease in VE‐cadherin expression, an increase in N‐cadherin expression, and a significant increase in BBB leakage. This effect was reversed by treatment with the MEK inhibitor U0126 in the rLCN2‐injected sham‐operated group of mice. Additionally, in BCAS mice treated with U0126, despite an increase in LCN2 expression, the MEK/ERK signaling pathway was inhibited, EndMT markers were reduced, and BBB integrity was restored compared to the BCAS 14‐day group (Figure 5I,J). These findings suggest that the MEK/ERK signaling pathway may serve as a critical mediating mechanism in LCN2‐driven EndMT.

### 3.8 Case–Control Study

To explore the potential of LCN2 as a specific biochemical marker for early warning and diagnosis of VCI, we correlated serum LCN2 levels with cognitive scores in VCI patients. Blood specimens from clinical VCI and control patients were collected, as well as the results of cognitive function assessment. PSM was employed to equilibrate the baseline characteristics, and Table 1 presents the fundamental characteristics of the patients both prior to and following matching. Post‐PSM, no significant differences were observed between the two groups concerning basic characteristics such as age, education, and body mass index (BMI). Additionally, the groups were well matched regarding cerebrovascular‐related risk factors, including smoking, alcohol consumption, hypertension, and diabetes mellitus. Our findings indicated that serum LCN2 levels were significantly elevated in the VCI group compared to the control group (*p* = 0.003). Further analysis of the correlation between serum LCN2 levels and MOCA scores in VCI patients revealed a significant negative correlation (Pearson's correlation coefficient *R* = −0.35, 95% CI = −0.5 to −0.17, *p* < 0.001), as illustrated in Figure S1. The analysis of inflammatory markers interleukin‐1β (IL‐1β) and tumor necrosis factor‐alpha (TNF‐α) demonstrated that TNF‐α was not significantly correlated with MOCA scores, whereas IL‐1β exhibited a significant positive correlation with MOCA scores. This finding suggests that IL‐1β and TNF‐α are not well suited to match MOCA scores, thereby underscoring the research potential of LCN2. Collectively, these results imply that LCN2 may serve as a promising biomarker for VCI.

**TABLE 1:** Basic information on patients and serum LCN2 content.

Varietes	Before PSM			After PSM		
	Control (*N* = 59)	VCI (*N* = 111)	*p*	Control (*N* = 53)	VCI (*N* = 53)	*p*
Age, mean ± SD	60 ± 11	65 ± 9	0.004*	62 ± 10	63 ± 9	0.677
Education, median (IQR)	12 (9, 15)	12 (9, 12)	0.015*	12 (9, 15)	12 (9, 13)	0.236
BMI, mean ± SD	24.10 ± 3.59	24.50 ± 3.28	0.563	23.85 ± 3.67	25.04 ± 3.21	0.078
Smoking, *n* (%)	24 (40.7)	51 (45.9)	0.51	22 (41.5)	26 (49.1)	0.435
Alcoholism, *n* (%)	15 (25.4)	39 (35.1)	0.195	13 (24.5)	21 (39.6)	0.096
Hypertension, *n* (%)	36 (61)	81 (73)	0.109	31 (58.5)	39 (73.6)	0.101
Diabetes, *n* (%)	21 (35.6)	43 (38.7)	0.687	19 (35.8)	25 (47.2)	0.237
MOCA, median (IQR)	27 (26, 29)	22 (19, 24)	< 0.001**	27 (26, 29)	22 (19, 24)	< 0.001**
LCN‐2, mean ± SD	48.0 ± 8.5	53.1 ± 11.2	0.007*	47.7 ± 8.5	53.6 ± 11.2	0.003*
TNF‐α, mean ± SD	58.48 ± 12.73	58.59 ± 16.62	0.963	57.94 ± 13.25	57.61 ± 18.86	0.917
IL‐1β, median (IQR)	26.16 (22.23, 36.53)	24.46 (21.04, 32.25)	0.133	26.16 (22.21, 36.21)	23.16 (20.43, 26.71)	0.023

*Note:* Patient clinical information before and after propensity score matching, including age, education, BMI, smoking, alcoholism, hypertension, diabetes, MOCA score, and LCN‐2/TNF‐α/IL‐1β content (ng/mL).

* *p* < 0.05.

** *p* < 0.001.

## 4 Discussion

VCI is becoming a major public health problem worldwide. In clinical practice, however, neurologists have limited therapeutic options [27, 28]. BBB damage usually propagates through various molecular cascades caused by CCH and induces a series of downstream events contributing to secondary brain injury, such as peripheral leukocyte infiltration [11]. Therefore, finding molecular markers of BBB dysfunction in the early stage of VCI is promising for prevention and treatment. Our research has identified LCN2 as a critical mediator in the disruption of the BBB during the early stages of CCH. Through the integration of transcriptomic analyses, clinical investigations, and molecular mechanism validation, we have demonstrated that LCN2 expression is significantly upregulated prior to the onset of cognitive impairment symptoms. This upregulation has been shown to induce EndMT via MEK/ERK phosphorylation. Furthermore, LCN2 is markedly elevated in the serum of patients with VCI and exhibits a significant negative correlation with the MOCA score, underscoring the therapeutic potential of targeting LCN2.

A recent cohort study has demonstrated a significant upregulation of LCN2 levels in the cerebrospinal fluid (CSF) of patients diagnosed with VaD [29]. Given the invasive nature of obtaining CSF through lumbar puncture [30], there is a compelling need to identify blood‐based biomarkers for early clinical screening. Our study confirmed that serum LCN2 levels are markedly elevated in patients with VCI compared to control subjects and showed a significant negative correlation with MOCA scores, indicating the potential of LCN2 as an early diagnostic marker. Nonetheless, the utility of LCN2 as an early warning indicator for VCI in individuals with cerebrovascular risk factors but without cognitive impairment necessitates further longitudinal studies.

Endothelial cells are a major component of the BBB, and triggering EndMT will cause vascular dysfunction, leading to BBB destruction [13]. Our observations indicate that LCN2 stimulation activates the MEK/ERK signaling pathway, thereby mediating EndMT, a process that can be reversed through the application of MEK inhibitors. Importantly, the present study bridges a fundamental gap in knowledge: although the MEK/ERK pathway is a well‐established mediator of epithelial‐mesenchymal transition (EMT) in cancer biology [31, 32], its involvement in the pathophysiology of VCI has not been adequately explored. Our findings uncover a previously unrecognized mechanistic link between LCN2 and cerebrovascular endothelial plasticity, suggesting a novel therapeutic target. Nonetheless, further comprehensive studies are required to fully elucidate these mechanisms, a task that presents considerable challenges. We anticipate that future research will address significant gaps in the current understanding.

During CCH, the endothelial cell inflammatory response due to hypoxia induces continuous recruitment of peripheral neutrophils to the cerebrovascular system, exacerbating BBB injury [33, 34]. Interestingly, LCN2, known as neutrophil gelatinase‐associated lipocalin (NGAL), is a key chemokine promoting neutrophil migration [35]. Previous studies have demonstrated that LCN2 promotes peripheral blood neutrophil infiltration in I/R injury [36, 37]. Our study further confirmed that LCN2 could promote the recruitment of peripheral neutrophils to the brain parenchyma in the early stage of VCI, which may play an essential role in the persistent neuroinflammatory state after CCH.

While the findings of this study enhance our understanding of the pathogenesis of VCI, the translation of these findings into clinical practice remains influenced by several factors. Firstly, the BCAS model employed induces uniform narrowing of the carotid arteries in mice, which may not accurately replicate the heterogeneous etiology observed in human VCI [38]. This includes conditions such as asymmetric carotid stenosis and comorbidities like hypertension and diabetes [39], which could impact the temporal and spatial expression patterns of LCN2. Furthermore, additional prospective studies are necessary to evaluate the potential of LCN2 as a biomarker for BBB dysfunction. Future research should also investigate the combined predictive value of LCN2 alongside other dementia‐related blood biomarkers, such as neurofilament light chain (NfL) [40, 41, 42], to enhance diagnostic accuracy. Despite these limitations, the study offers promising directions for future research aimed at predicting BBB injury and identifying high‐risk VCI patients.

## Author Contributions

Conception and design: Yuchao Li and Qianqian Nie. Administrative support: Xiaoying Bi. Provision of study materials or patients: all authors. Collection and assembly of data: Yuchao Li, Qianqian Nie, and Liren Zhang. Data analysis and interpretation: Yuchao Li and Qianqian Nie, Liren Zhang, and Zhengsheng Gu. Manuscript writing: Qianqian Nie; final approval of manuscript: all authors.

## Conflicts of Interest

The authors declare no conflicts of interest.

## Supporting information


**Figure S1.** Correlation analysis of serum LCN2 content and MOCA score in VCI patients.

## Data Availability

The data that support the findings of this study are openly available in Gene Expression Omnibus at https://www.ncbi.nlm.nih.gov/gds/?term=, reference number GSE22063.
