# Supplementary material for: Lipocalin 2 Facilitates the Initial Compromise of the Blood–Brain Barrier Integrity in Chronic Cerebral Hypoperfusion
Source: CNS Neurosci Ther. 2025 May 14;31(5):e70438. doi: 10.1111/cns.70438 (PMC12076118; doi:10.1111/cns.70438)
Supplement: Supplementary file 1 — Figure S1. Correlation analysis of serum LCN2 content and MOCA score in VCI patients. [file CNS-31-e70438-s001.docx]

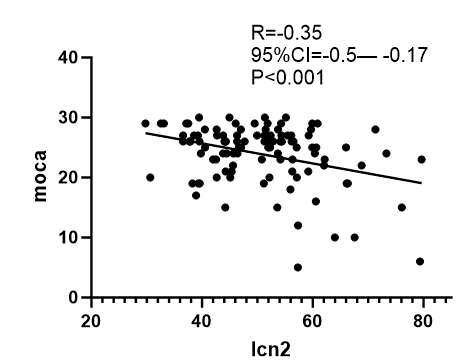


Supplementary Figure 1: Correlation analysis of serum LCN2 content and MOCA score in VCI patients.(Pearson’s R=-0.35, 95%CI[-0.5,-0.17], P<0.001)
